# From Fish Oil to Resolution: A Narrative Review on the Potential of SPM-Enriched Marine Oil for Exercise-Induced Muscle Damage Recovery

**DOI:** 10.3390/nu17122014

**Published:** 2025-06-16

**Authors:** Leticia C. de Souza, Jose M. Moris, Paul M. Gordon, Jeffery L. Heileson, LesLee K. Funderburk

**Affiliations:** 1 Department of Health, Human Performance, and Recreation, Baylor University, Waco, TX 76706, USA; paul_m_gordon@baylor.edu (P.M.G.); jeffery.l.heileson.mil@army.mil (J.L.H.); leslee_funderburk@baylor.edu (L.K.F.); 2 Cerebrovascular & Cognition Laboratory, Department of Health Science, Texas A&M International University, Laredo, TX 78041, USA; jose.moris@tamiu.edu; 3 Nutrition Services Division, Walter Reed National Military Medical Center, Bethesda, MD 20889, USA; 4 Department of Human Sciences and Design, Baylor University, Waco, TX 76706, USA

**Keywords:** nutritional supplements, injury prevention, skeletal muscle function, athletic performance, physical performance, dietary recommendations, neutrophil infiltration, macrophage polarization

## Abstract

Exercise-induced muscle damage (EIMD) initiates an inflammatory response that is essential for tissue repair. However, when prolonged or excessive, this response can impair recovery and muscular performance. Specialized pro-resolving mediators (SPMs), derived from the metabolism of omega-3 (*n*-3) polyunsaturated fatty acids (PUFAs), facilitate the resolution of inflammation without causing immunosuppression. Evidence from preclinical studies indicates that SPM administration accelerates muscle repair and functional recovery by enhancing the clearance of apoptotic cells, suppressing pro-inflammatory signaling and modulating macrophage polarization. However, translation to human applications remains limited as commercially available SPM-enriched marine oils do not contain active SPMs but rather their monohydroxylated precursors, including 14-Hydroxy-Docosahexaenoic Acid (14-HDHA), 17-Hydroxy-Docosahexaenoic Acid (17-HDHA), and 18-Hydroxy-Eicosapentaenoic Acid (18-HEPE) in addition to low doses of the *n*-3 PUFAs eicosapentaenoic acid (EPA) and docosahexaenoic acid (DHA). Furthermore, the variable increases in circulating SPM concentrations as a result of dietary intake of EPA and DHA, whether from fish or fish oil supplements, and the wide diversity of SPM molecules (many of which remain under investigation), highlight the complexity of their structural and functional networks. While advances in lipidomics have identified SPMs and their pathway intermediates in human biological samples, further research is needed to determine optimal dosing strategies, delivery mechanisms, and the real impact of SPM-enriched marine oil on athletic performance and recovery. This narrative review examines the biological rationale and current evidence surrounding SPM-enriched marine oil supplementation and its potential to enhance muscle recovery following EIMD. By synthesizing findings from preclinical and human studies, the potential of SPM-enriched supplementation as a novel tool for optimizing performance recovery in athletic populations is reviewed to inform future research directions.

## 1. Introduction

Athletes and physically active individuals can experience muscle soreness and performance decrements following intense training, particularly after high-force eccentric exercise [1]. Unlike concentric or isometric contractions, eccentric movements require muscles to lengthen under load while recruiting fewer motor units [2], thereby placing greater mechanical stress on individual muscle fibers. A heightened mechanical strain increases muscles’ susceptibility to microscopic structural damage and triggers a localized inflammatory response [3]. Edema, pain, restricted range of motion, and reduced force generation are examples of symptoms that emerge following an inflammatory response [4]. While inflammation is needed for muscles to adapt, there is a temporary muscular impairment that hinders athletic performance. Factors such as exercise intensity, training history, and the extent of muscle damage determine the duration of symptomology and the associated athletic performance decline, with symptoms typically peaking within 24 to 48 h post-exercise and persisting for up to 10 days in more severe cases [1].

Because athletes and fitness enthusiasts frequently train under intense loads to meet the physical demands of their sport, several strategies, ranging from therapeutic interventions to dietary supplementation, have been explored to alleviate muscle soreness, reduce the inflammatory response, and facilitate recovery to maintain athletic performance [5]. Among these, supplementation with the omega-3 (*n*-3) polyunsaturated fatty acids (PUFAs) eicosapentaenoic acid (EPA) and docosahexaenoic acid (DHA) has shown recovery benefits due to its anti-inflammatory properties [6,7,8,9,10]. Recent evidence, however, suggests that the primary anti-inflammatory effects of EPA+DHA are not solely attributable to the fatty acids themselves but are largely a result of their metabolization into specialized pro-resolving mediators (SPMs) [11,12,13,14]. These SPMs are enzymatically synthesized from EPA and DHA through tightly regulated pathways. The conversion occurs locally at sites of inflammation and gives rise to several families of bioactive lipid mediators, termed resolvins, protectins, and maresins. In general, SPMs are a superfamily of “highly specialized” molecules that play a critical role in coordinating the resolution of inflammation [15,16].

SPMs were discovered nearly 20 years ago as key regulators of the transition from inflammation initiation to resolution and tissue repair [16]. They facilitate neutrophil clearance, suppress inflammatory cytokine production, and promote macrophage polarization from a pro-inflammatory to an anti-inflammatory phenotype [15,16]. Preclinical evidence demonstrates that daily systemic administration of Resolvin D1 (RvD1), an SPM derived from the metabolization of DHA, enhances myofiber regeneration and muscle strength recovery following an acute muscle injury compared to untreated injured controls [17]. This suggests that enhancing the endogenous production of SPMs may help mitigate the prolonged functional deficits associated with exercise-induced muscle damage (EIMD), although randomized controlled trials in humans are needed.

Since the identification of SPMs as regulators of inflammation [16], interest in their therapeutic applications has grown, particularly in the form of SPM-enriched marine oil emulsions or dietary supplements [18,19,20,21,22,23,24,25]. While conventional fish oil supplements provide EPA and DHA, the precursors for SPM biosynthesis, their enzymatic conversion into bioactive SPMs is context-dependent [26] and may be constrained by rate-limiting steps involving cyclooxygenase (COX), lipoxygenase (LOX), and cytochrome P450 enzymes [26]. As a result, although regular fish oil supplementation increases circulating SPM concentrations [27,28,29,30,31], these increases are not uniform across all SPM families and may be less pronounced when enzymatic activity is insufficient, when dietary omega-6 polyunsaturated fatty acids (*n*-6 PUFAs) intake is high (therefore resulting in an elevated *n*-6: *n*-3 PUFA ratio), or when SPM synthesis is impaired [32]. To address these potential limitations, SPM-enriched marine oils are extracted from marine organisms rich in long-chain *n*-3 PUFAs and contain high concentrations of SPM pathway intermediates compared to conventional fish oil. These pathway intermediates include 18-Hydroxy-Eicosapentaenoic Acid (18-HEPE), 17-Hydroxy-Docosahexaenoic Acid (17-HDHA), and 14-Hydroxy-Docosahexaenoic Acid (14-HDHA), which are one enzymatic step away from bioactive SPMs. Although these intermediates are not bioactive SPMs, their structural similarity and metabolic proximity to downstream SPMs suggest that supplementation may enhance the bioavailability of SPMs and promote anti-inflammatory cascades associated with muscle recovery [13,16,17]. This may allow SPM-enriched formulations to bypass one or more rate-limiting enzymatic steps required for EPA and DHA conversion to SPMs, although further research is needed to confirm this hypothesis. Interestingly, while only a few studies have explored the effects of SPM-enriched marine oil in humans, none have investigated its impact on recovery from EIMD. To date, human investigations have predominantly focused on clinical populations with chronic or inflammatory conditions rather than healthy, physically active individuals [18,20,21,22,23,24,25,33].

This narrative review has three primary aims. First, we present an overview of the key mechanisms through which *n*-3 PUFA supplementation enhances recovery from unaccustomed, strenuous exercise, establishing a foundation for understanding the role of SPMs in inflammation resolution. Second, we examine evidence from in vivo preclinical studies using the administration of bioactive SPMs—particularly those involving soft tissue injury models—and human trials investigating naturally occurring concentrations of either SPMs or their pathway intermediates and their roles in resolving inflammation. Third, we comprehensively assess existing research exploring the effects of SPM-enriched marine oil supplementation in humans, highlighting knowledge gaps that warrant further investigation. This review explores the therapeutic potential of SPM-enriched marine oil in enhancing recovery following strenuous exercise. The goal is to inform future research directions and support the development of tailored sports nutrition strategies aimed at optimizing recovery and athletic performance.

## 2. Materials and Methods

A literature search was conducted using Medline (PubMed) and Google Scholar. For preclinical studies, relevant articles were identified using the search terms “myofiber regeneration”, “muscle stem cells”, “muscle regeneration”, “tissue homeostasis”, “pain”, and “recovery”, in combination with “resolvin” or “lipid mediators”. For human studies investigating SPM-enriched marine oil, the search included the following terms: “SPM-enriched marine oil”, “enriched marine oil”, “SPM-enriched marine lipid fraction”, “SPMs-enriched oil”, “specialized pro-resolving lipid-mediator-enriched marine oil”, “marine oil supplement”, “specialized pro-resolving mediator-enriched oil”, and “SPM-enriched fish oil”, along with outcome-related terms such as “plasma SPMs”, “serum SPMs”, “plasma lipid mediators”, “serum lipid mediators”, “inflammatory biomarkers”, “immune cell response”, and “pain”.

In addition, observational, case-control, retrospective, and randomized controlled studies published in English that investigated physiological concentrations of SPMs and their precursors were included. Relevant narrative and systematic reviews were also incorporated to provide broader context on inflammation resolution processes and the effects of omega-3 PUFAs and SPMs on skeletal muscle recovery, inflammation, and pain outcomes.

## 3. Omega-3 PUFAs and Specialized Pro-Resolving Mediators in Inflammation and Recovery

### 3.1. Role of Omega-3 Polyunsaturated Fatty Acids in Inflammation and Recovery

The initial recognition of the benefits of *n*-3 PUFAs derived from studies of Inuit populations, whose fish-rich diet, high in EPA and DHA, correlated with lower rates of cardiovascular disease [34]. Beyond cardiovascular benefits, EPA and DHA play crucial roles in reducing inflammation through their incorporation into cellular membranes [35]. As a result of increased dietary intake of EPA and DHA, these fatty acids are incorporated into phospholipids in cellular membranes and influence membrane integrity, fluidity, and function. These changes play a vital role in the response to physiological stress and maintenance of cellular homeostasis by influencing signaling pathways, as well as gene and protein expression [35,36,37].

The composition of cellular membranes is important because it affects how cells respond to stress. Notably, the production of inflammatory lipid mediators is influenced by the abundance of either *n*-3 or *n*-6 PUFAs in cellular membranes. For example, EPA and arachidonic acid (AA), an *n*-6 PUFA also released from cellular membranes, have shared competition for metabolic enzymes, including COX and LOX [38]. If AA is the leading metabolic compound, the outcome is a pro-inflammatory lipid-mediated physiological stress via the production of prostaglandins (PGs), thromboxanes (TXs), and leukotrienes (LTs) (e.g., four-series leukotrienes and two-series prostanoids [PGI2]) [38], whereas EPA metabolism yields low-grade inflammatory mediators (five-series leukotrienes and three-series prostanoids [PGI3]) [37]. This enzymatic competition represents a key mechanism in reducing inflammation because the shift from AA metabolism to EPA results in a blunted activation of nuclear factor kappa beta (NF-κB) and a subsequent mitigation in pro-inflammatory cytokine and chemokine release. This competition particularly highlights the importance of maintaining a proper *n*-6: *n*-3 PUFA ratio, denoting that *n*-3 PUFA availability should be prioritized.

Interestingly, while an *n*-6: *n*-3 PUFA ratio of 4:1 is generally recommended, the typical Western diet often reaches levels as high as 15:1 [39]. This imbalance has led to the emerging interest in *n*-3 PUFA supplementation as an anti-inflammatory strategy. Of note, while EPA and DHA can be synthesized endogenously from alpha-linoleic acid (ALA), which is the only recognized essential *n*-3 PUFA, the conversion rate of ALA to EPA and DHA is extremely low (less than 5%) [40]. Consequently, the dietary intake of EPA and DHA is necessary to meet basic physiological needs.

Given the critical roles of EPA and DHA in modulating inflammation and improving the *n*-6: *n*-3 PUFA balance, fish oil supplementation has been demonstrated to enhance recovery from strenuous exercise by preserving strength [6], reducing muscle soreness [6,7,8,41], accelerating the restoration of jump performance [6,8], and reducing inflammatory biomarkers [7,41]. A recent systematic review reported that fish oil supplementation promotes recovery from EIMD by reducing inflammatory biomarkers [9]. However, a downside of *n*-3 PUFA supplementation for enhanced recovery and athletic performance includes the requirement of sustained intake. This is the case because the efficacy of *n*-3 PUFA supplementation seems to mostly depend on its gradual incorporation into muscle membranes. Significant increases in skeletal muscle fatty acid composition generally require a minimum of four weeks [9,42]. This recommendation is based on an investigation by McGlory et al., which obtained serial muscle biopsies from the vastus lateralis of healthy young males to track EPA and DHA incorporation into skeletal muscle tissue [42]. Daily intake of 5 g of fish oil (4.4 g EPA + DHA, including 3.5 g EPA and 0.9 g DHA) resulted in detectable changes in muscle phospholipids by week two, with further rises at week four, a contrasting finding compared to blood lipid changes, which occurred as early as week one [42]. Therefore, this time-dependent incorporation may explain why shorter supplementation periods (<4 weeks) often fail to improve muscle recovery markers following strenuous exercise [43,44]. In contrast, prolonged supplementation, lasting four to ten weeks, has been demonstrated to promote maximal incorporation into skeletal muscle membranes and enhance recovery outcomes following strenuous exercise [6,7,8,9].

Despite these findings, prolonged supplementation does not guarantee functional improvements. For instance, a study in recreationally active women reported no improvements in leg torque or specific force, despite eight weeks of supplementation (5 g/day; 2.97 g EPA + 2.03 g DHA) initiated four weeks prior to immobilization and continued through two weeks of immobilization and two weeks of recovery [45]. Notably, muscle volume loss was attenuated compared to controls, and phospholipid EPA+DHA content reached saturation by week six, indicating that the supplement effectively incorporated into skeletal muscle tissue. However, while these findings suggest that structural preservation is achievable through supplementation, membrane incorporation alone may not be sufficient to elicit functional benefits.

While immobilization and EIMD differ in nature, both elicit transient functional impairments and regenerative demand. Therefore, the protective effects of *n*-3 PUFAs (e.g., membrane remodeling, stimulation of muscle protein synthesis, anabolic signaling, and anti-inflammatory actions) may also facilitate recovery from EIMD. Given that maximal membrane incorporation alone may not fully explain functional benefits, recent attention has turned to alternative pathways by which *n*-3 PUFAs may exert their effects. One such area of interest involves a large class of downstream lipid mediators, particularly bioactive oxylipins, such as specialized pro-resolving mediators (SPMs) due to their unique roles in inflammation resolution and repair.

### 3.2. Oxylipins: The Broader Family of Lipid Mediators

The anti-inflammatory actions of EPA and DHA are mediated, in part, by their conversion into oxygenated lipid mediators known as oxylipins. These compounds are synthesized from PUFAs, including AA, EPA, DHA, linoleic acid (LA), and ALA, via COX, LOX, and cytochrome P450 enzymatic pathways. Oxylipins exist in either free, biologically active forms or esterified within membrane phospholipids, where they may influence membrane function or serve as stored precursors for localized conversion into active mediators. 

Oxylipins encompass structurally and functionally diverse compounds, such as PGs, leukotrienes, thromboxanes, epoxyeicosatrienoic acids (EETs), and hydroxyeicosatetraenoic acids (HETEs), which regulate inflammation, vascular tone, pain, and tissue repair [46]. Their functional properties are influenced by PUFA origin: AA- and LA-derived oxylipins (from *n*-6 PUFAs) are generally more pro-inflammatory, whereas those from EPA and DHA (*n*-3 PUFAs) are typically less potent and promote anti-inflammatory and pro-resolving actions. 

Although dietary PUFA intake shapes oxylipin profiles, the relationship is complex and non-linear. As reviewed by Gabbs et al., enzymatic specificity, phospholipid incorporation, and metabolic turnover contribute to significant individual variability. For instance, ALA may be converted to oxylipins at rates up to ten times greater than LA, AA, or EPA [47]. Due to this complexity, direct quantification of oxylipins is recommended for understanding the physiological effects of dietary fats [46]. Among this broad class, a distinct subset of oxylipins has emerged as a key regulator of inflammation resolution: the specialized pro-resolving mediators (SPMs).

### 3.3. The Discovery of Specialized Pro-Resolving Mediators

While *n*-3 PUFA supplementation has been reported to improve muscle recovery following prolonged supplementation periods [9,45], novel evidence suggests that *n*-3 PUFAs primarily exert their anti-inflammatory effects through their enzymatically oxygenated metabolites, referred to as SPMs [14,15,16]. The term SPMs was identified by Dr. Serhan in 2008, who challenged the traditional view of inflammation as a passive process. Dr. Serhan’s team hypothesized that specific, previously unidentified mediators must actively drive the resolution phase of inflammation [48]. Thus, using liquid chromatography and tandem mass spectrometry (LC-MS/MS), his team demonstrated that, in the presence of aspirin, EPA [49] and DHA [48] irreversibly modify COX-2 activity, thereby producing a new class of oxygenated lipid mediators termed resolvins. The identified E-series resolvins originated from EPA [49], while D-series resolvins originated from DHA [48]. These molecules, first identified in inflammatory exudates of preclinical models, attenuated cytokine production and reduced leukocyte infiltration in acute inflammation [48]. Subsequent research identified additional families of lipid mediators, including lipoxins derived from AA, protectins (DHA-derived), and maresins (DHA-derived), who also fall under the umbrella term SPMs due to their specialized roles in inflammation resolution [16]. The resolvins formed from aspirin have a different stereochemical structure from regular resolvins and are termed aspirin-triggered SPMs. Generally, each SPM family includes several molecules, and their roles and quantification methods are still under investigation [50,51].

### 3.4. Roles of Specialized Pro-Resolving Mediators in Inflammation Resolution

EIMD disrupts sarcomeres and damages the sarcolemma, leading to increased vascular permeability [16]. The resulting vascular leakage triggers a localized inflammatory response aimed at eliminating the cause of injury and sets the stage for tissue restoration [11,13,52]. Because SPMs orchestrate the resolution of this inflammatory response, understanding their function requires recognizing that inflammation consists of two interconnected phases: initiation and resolution, where the signaling events that drive inflammation initiation also program its termination, a process termed inflammation resolution.

Following EIMD, the initiation phase of inflammation is characterized by the rapid production of lipid mediators and pro-inflammatory cytokines that coordinate immune cell recruitment. Eicosanoids, synthesized from AA (e.g., PGs, LTs, and TXs) via COX enzymes, stimulate the release of pro-inflammatory cytokines, such as tumor necrosis factor-alpha (TNF-α) and interleukins [53]. The resulting pro-inflammatory microenvironment promotes the infiltration of polymorphonuclear neutrophils, which migrate (post-EIMD) to the damaged area within an hour to clear debris via phagocytosis [54]. Subsequently, pro-inflammatory (M1) macrophages are recruited to amplify the inflammatory response. Although early neutrophil activity is essential for initiating tissue repair by clearing debris and recruiting macrophages [55], sustained neutrophil presence promotes excessive production of reactive oxygen species (ROS) and proteases, further exacerbating tissue damage [54]. During the early phase of inflammation initiation, localized tissue disruption, increased vascular permeability, and immune cell infiltration underlie the cardinal signs of inflammation, including heat, swelling (edema), redness, pain, and, in severe cases, loss of function. The magnitude of these symptoms increases with the extent of muscle damage, which is reflected by elevated concentrations of muscle damage markers, such as creatine kinase, myoglobin, and pro-inflammatory cytokines [56]. Importantly, the resolution of this response, coordinated by lipid mediators such as SPMs, is critical for preventing secondary injury, promoting effective muscle regeneration, and achieving a timely alleviation of clinical symptoms.

As inflammation progresses, resolution emerges as an active and tightly regulated process driven by the increasing production of SPMs. SPMs limit neutrophil infiltration and promote macrophage polarization from a pro-inflammatory to a reparative, pro-resolving (M2) phenotype [17,57]. The gradual change in the relative abundance of lipid mediators, where early *n*-6-derived mediators (e.g., PGs, LTs, and TXs) are progressively complemented by rising levels of *n*-3-derived SPMs, is referred to as the lipid mediator “class switch”. This dynamic interplay has been characterized in both humans [53,58] and murine models [59].

For instance, in a preclinical model of acute muscle injury, pro-inflammatory mediators, such as leukotriene B_4_ (LTB_4_), prostaglandin E_2_ (PGE_2_), and prostaglandin F2α (PGF2α), dominated the early inflammatory phase (days 0–2) [59]. However, by days 2 and 8, concentrations of SPMs and their precursors, including Resolvin D2 (RvD2) and resolvin E1 (RvE1), increased, coinciding with a rise in pro-resolving macrophages [59]. Importantly, while *n*-6-derived lipid mediators dominate the early inflammatory response, *n*-3-derived SPMs are already present during this phase and may contribute to early resolution signaling. Conversely, certain *n*-6-derived mediators, particularly lipoxins synthesized via the LOX pathway from AA, also exhibit pro-resolving and tissue remodeling functions. Collectively, these findings support a model of temporal coordination rather than a binary shift, where the relative abundance and timing of *n*-3 and *n*-6 oxylipins orchestrate both the initiation and resolution phases of inflammation.

Additionally, pro-resolving macrophages enhance efferocytosis, the process of apoptotic cell clearance, thereby preventing the release of toxic intracellular content and limiting secondary tissue damage, while SPMs suppress pro-inflammatory cytokine production [60,61]. Additionally, pro-resolving macrophages also secrete factors such as insulin-like growth factor (IGF-1) [62] that support satellite cell activation, proliferation, and differentiation, processes necessary for myofiber regeneration.

Consistent with findings in murine models, similar time-dependent patterns of inflammatory-mediated production have been observed in humans following intense, unaccustomed, resistance exercise. After maximal concentric and eccentric contractions, elevated intramuscular concentrations of AA-derived pro-inflammatory mediators (e.g., PGE_2_, PGF_2_α, TXB_2_) have been detectable within two hours post-exercise [53,58]. SPMs have also been identified both in the early phase (0–3 h) [53,58] and later recovery phases (24 h) [58], indicating a time-response coordinated activation of resolution pathways [58]. Nevertheless, no studies have yet characterized SPM dynamics beyond 24 h following exercise in humans, leaving the longer-term kinetics of resolution mediators poorly understood.

**Figure 1:**
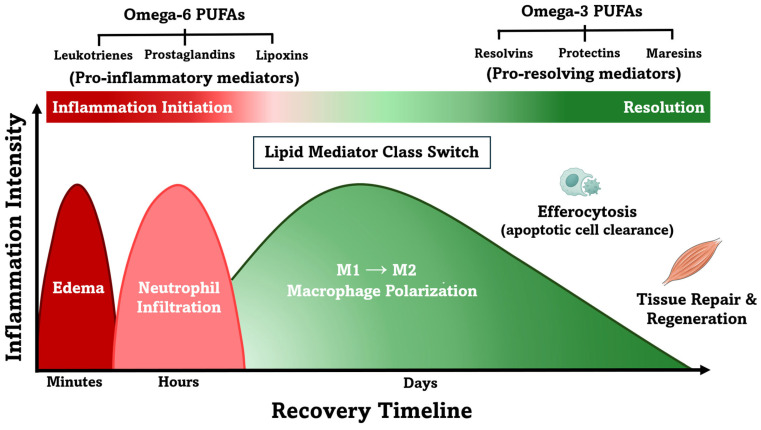
Inflammation initiation and resolution following exercise-induced muscle damage (EIMD). This figure illustrates key immune cell transitions and the lipid mediator class “switch” from pro-inflammatory to pro-resolving signaling. Omega-6-derived mediators (leukotrienes, prostaglandins) dominate early inflammation, while omega-3-derived specialized pro-resolving mediators, including resolvins, protectins, and maresins, facilitate resolution. Although derived from omega-6 fatty acids, lipoxins are classified as early pro-resolving mediators and promote neutrophil clearance and macrophage phenotype change. This figure was re-created and adapted based on concepts and visuals from Markworth et al. (2016) [13] and Sansbury and Spite (2016) [66]. Macrophage and muscle fiber illustrations are attributed to Smart Servier Medical Art, which is licensed under Creative Commons Attribution 3.0 Unported License.

In addition, the suggested molecular events orchestrated by lipid mediators coincide with the clinical progression of recovery following EIMD in humans [11,13,52]. Inflammatory responses are typically observed immediately after exercise, peaking within the first 48 h, and are followed by attenuated EIMD symptoms around 72 h, marked by reduced muscle soreness and partial restoration of strength [1,6,8,63]. Notably, these clinical changes coincide with the transition from the initiation to the resolution phase of inflammation. While timely SPM activity promotes homeostasis and tissue repair, impaired resolution, whether due to an exaggerated initial inflammatory response or a failure to activate pro-resolving pathways, can prolong immune cell infiltration, sustain pro-inflammatory cytokine release, and increase the risk of fibrosis and chronic inflammation [13,64]. Aside from intrinsic limitations in resolution pathways, external influences, such as non-steroidal anti-inflammatory drug (NSAID) use, can delay muscle recovery and blunt exercise-induced adaptations. For example, high doses of NSAIDs blunt strength gains and hypertrophic adaptations to resistant training, which may impair athletic performance over time [65]. Figure 1 illustrates the role of SPMs in coordinating a controlled inflammatory response, analogous to a restoration team that prevents excessive structural damage and prepares the tissue microenvironment for regeneration following the initial “demolition” or injury.

### 3.5. Inflammation Resolution vs. Suppression: Distinct Mechanisms of SPMs and NSAIDs

Given the critical role of SPMs in orchestrating muscle recovery, it is important to distinguish their mechanisms of action from those of conventional anti-inflammatory strategies, such as NSAIDs. NSAIDs are widely consumed drugs, especially among athletes and physically active individuals [67]. However, these drugs reduce inflammation by blocking COX enzyme activity, thereby suppressing the synthesis of PGs, including PGF2α and PGE2, key regulators of muscle hypertrophy and repair [68,69]. Although NSAIDs provide short-term pain relief by inhibiting PG production, prolonged use impairs muscle adaptation and hypertrophy [58,70]. Importantly, PGs not only initiate inflammation but also serve as precursors for SPM synthesis. Thus, NSAIDs can disrupt both the initiation and resolution phases of inflammation by suppressing pro-inflammatory and pro-resolving macrophage activity [71]. The negative effects of NSAIDs on inflammation resolution have been demonstrated in healthy humans, where ibuprofen intake before unaccustomed resistance training suppressed endogenous SPM production [58] and compromised strength and hypertrophic adaptions [65]. In contrast, SPMs resolve inflammation through mechanisms that preserve COX enzyme activity, promoting both anti-inflammatory and pro-reparative actions without inhibiting beneficial inflammatory signaling. Given these distinct mechanisms, SPMs present a promising therapeutic target for optimizing tissue recovery by restoring homeostasis rather than merely suppressing symptoms.

### 3.6. Synthesis of Specialized Pro-Resolving Mediators from Omega-3 PUFAs

**Figure 2:**
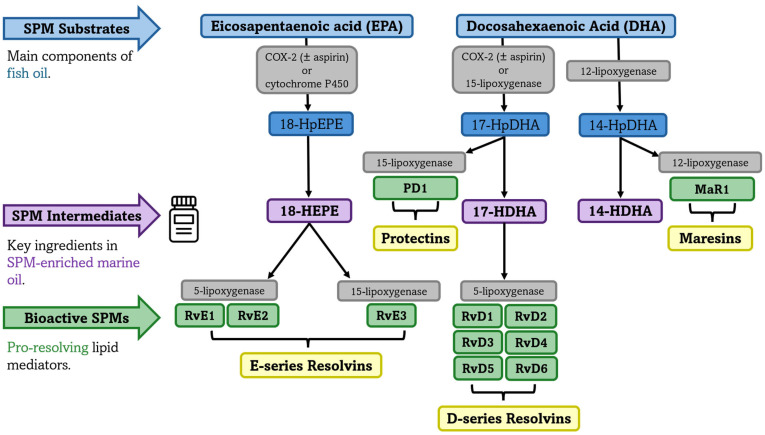
Biosynthesis of specialized pro-resolving mediators (SPMs) derived from eicosapentaenoic acid (EPA) and docosahexaenoic acid (DHA). The distinct families of SPMs are displayed in yellow boxes, including E-series resolvins (RvE1, RvE2, and RvE3), derived from EPA, and D-series resolvins (RvD1, RvD2, RvD3, RvD4, RvD5, and RvD6), protectins (PD1), and maresins (MaR1), derived from DHA. SPM synthesis is organized into three hierarchical levels: (1) SPM substrates (light blue); (2) SPM pathway intermediates (purple), which serve as monohydroxylated precursors to SPMs and are the bioactive ingredient in SPM-enriched marine oil supplements; and (3) bioactive, end-stage SPMs (green). Enzymatic conversions are indicated in gray boxes. Cyclooxygenase (COX-2; ± aspirin) indicates that both native and aspirin-acetylated COX-2 can participate in SPM biosynthesis, leading to stereochemically distinct mediators. Arrows denote the direction of each enzymatic conversion. Abbreviations: COX-2 (cyclooxygenase-2); HpEPE (hydroperoxyeicosapentaenoic) acid; HEPE (hydroxy-eicosapentaenoic acid); HpDHA (hydroperoxydocosahexaenoic acid); HDHA (hydroxy-docosahexaenoic acid); Rv (Resolvin); PD1 (Protectin D1); MaR1 (Maresin 1).

SPMs are a unique class of lipid mediators that, unlike their parent compounds EPA and DHA, are not obtained directly from dietary sources. Instead, SPMs are synthesized locally at sites of inflammation through the enzymatic conversion of these essential fatty acids. This process involves specific enzymes, including COX, LOX, and cytochrome P450 monooxygenase [32], that metabolize EPA and DHA into SPM pathway intermediates. These intermediates, including 18-Hydroxy-Eicosapentaenoic Acid (18-HEPE) from EPA and 17-Hydroxy-Docosahexaenoic Acid (17-HDHA) and 14-Hydroxy-Docosahexaenoic Acid (14-HDHA) from DHA, are then converted into four main families of SPMs: EPA-derived E-series resolvins and DHA-derived D-series resolvins, maresins, and protectins. Each family contains several distinct molecules with specific functions. For instance, 18-HEPE leads to the E-series resolvins E1, E2, and E3 (RvE1, RvE2, RvE3), while 17-HDHA yields the D-series resolvins (including RvD1, RvD2, RvD3, RvD4, RvD5, and RvD6) and 17- Hydroxyperoxydocosahexaenoic acid (17-HpDHA) gives rise to protectins (e.g., PD1). In parallel, 14-Hydroxyperoxydocosahexaenoic acid (14-HpDHA) gives rise to maresins (e.g., MaR1) [26,72,73]. Figure 2 illustrates the biosynthesis of SPMs derived from the *n*-3 PUFAs EPA and DHA.

SPM production is triggered in response to acute inflammation, infection, injury, or eccentric, strenuous, or unaccustomed exercise [17,53,58,59,74], which ensures these mediators are produced precisely when and where they are needed. For example, during inflammatory conditions, the local enzymatic conversion of 18-HEPE and 17-HDHA to their respective E- and D-series SPMs is significantly enhanced within inflamed tissues [26,72,75], as observed in arthritis patients, where synovial fluid shows higher conversion efficiency compared to plasma [26]. Similarly, plasma SPM concentrations in patients with arthritis have been found to be elevated in comparison to healthy volunteers, as well as the conversion from SPM intermediates to bioactive SPMs [26].

However, a distinctive feature of SPMs is their rapid metabolism and inactivation, which occurs within minutes to hours depending on their structure and administration route [76]. For example, the presence of RvE1 has been reported in human serum 0–1 h following a bout of unaccustomed, eccentric exercise, while RvD1 has been reported within 2–3 h following exercise, with the latter remaining elevated following 24 h of recovery [58]. However, the biosynthesis of specific mediators can occur at different timepoints depending on the severity of the muscle injury inflicted and the time course of the inflammatory response [13]. While this time-dependent regulation ensures precise control of inflammation resolution, it also presents challenges for clinical applications due to their short biological half-life and chemical instability. The rapid metabolism of SPMs distinguishes itself from EPA, DHA, and their intermediates, which can accumulate in tissues and exert prolonged effects [61,75].

## 4. Therapeutic Applications of Specialized Pro-Resolving Mediators (SPMs)

### 4.1. Therapeutic Applications of SPMs in In Vivo Preclinical Models

SPMs derived from *n*-3 PUFAs demonstrate therapeutic potential across preclinical models of muscle injury, arthritis, and periodontal disease through multiple mechanisms, including the promotion of macrophage polarization, suppression of pro-inflammatory cytokines, and enhancement of tissue regeneration [17,59,77,78]. In a murine model of acute muscle injury, daily systemic administration of RvD1 (100 ng), initiated 5 min post-injury, increased myofiber cross-sectional area by day 5 and restored 15% of muscle strength by day 14, primarily through type IIb fiber (functionally comparable to humans’ type IIx) hypertrophy [17]. These regenerative effects were accompanied by reductions in interleukin 6 (IL-6) and TNF-α concentrations at 24 h and a ~40% decrease in neutrophil infiltration at 72 h compared to placebo-treated injured controls. RvD1 also promoted macrophage polarization, as indicated by elevated arginase-1 and CD163+ expression, and accelerated satellite cell differentiation, reflected by reduced paired box 7-positive (Pax7+) cell density and increased myogenin expression, by day 5 [17]. Notably, these findings suggest that RvD1 facilitates the progression of satellite cells from a quiescent or proliferative state, marked by Pax7+ expression, toward differentiation and fusion with regenerating myofibers [17].

In contrast to sustained SPM administration, functional outcomes following single-dose interventions have also been explored. In a murine model of impaired muscle regeneration (satellite cell depletion), Giannakis et al. reported that a single intramuscular injection of RvD2 (4 µg/kg), administered three days post-injury, improved force recovery (by ~50%) and muscle mass (by ~17%) at days 8 and 14 post-injury compared to saline-treated injured controls [59]. These findings suggest that isolated SPM administration enhances regenerative capacity, especially in the context of impaired endogenous repair.

Additionally, both SPM intermediates and bioactive SPMs have demonstrated analgesic properties in clinical models. Huang et al. and Lima-Garcia et al. examined the effects of 17(R)-Hydroxy-Docosahexaenoic acid (17[R]-HDHA), an aspirin-triggered SPM intermediate formed by COX-2 mediated oxygenation of DHA, in rat models of osteoarthritis- and arthritis-induced inflammation [79,80]. In the study by Huang et al., a single 300 ng dose of 17[R]-HDHA provided pain relief within an hour, with beneficial effects persisting for up to six hours [79]. Moreover, daily administration over two weeks sustained analgesic benefits for up to seven days following treatment cessation, correlating with increased plasma RvD2 and enhanced detectability of RvD1 [79]. Similarly, Lima-Garcia et al. demonstrated that treatment with 300 ng/day of 17[R]-HDHA or Aspirin-Triggered RvD1 (AT-RvD1) reduced joint stiffness and pain and inhibited the production of pro-inflammatory cytokines, including TNF-α and interleukin 1 beta (IL-1β), by blocking the activation of NF-kβ [80]. Although the authors noted that the metabolism and pharmacokinetics of these lipids remain incompletely characterized, in their study, 17[R]-HDHA was administrated once daily for five days, whereas AT-RvD1 was administered twice daily for four days [80].

In addition to systemic and intramuscular delivery, localized application strategies have been investigated to optimize SPM efficacy in tissue repair. Hasturk et al. demonstrated that topical application of RvE1 (4 µg/site, three times per week for six weeks) reduced systemic biomarkers of inflammation [IL-1β and C-Reactive Protein (CRP)] and restored 95% of lost bone tissue, along with reported soft tissue regeneration, in a rabbit model of periodontitis [77]. Given that periodontitis is driven by neutrophil-mediated tissue destruction (which results in the loss of connective tissue and bone supporting the teeth), these findings emphasize SPM’s ability to limit neutrophil infiltration without suppressing beneficial inflammatory responses that promote inflammation resolution.

Similarly, Turner et al. investigated the effects of slow-release, localized delivery of hydrogel-based AT-RvD1 (100 ng/day) for 24 h. The hydrogel delivery resulted in an 86% restoration of maximal isometric torque (by day 25)in a murine model of volumetric muscle loss [78]. Interestingly, despite adequate DHA and EPA availability, the injury model exhibited impaired endogenous conversion of 17-HDHA. The hydrogel formulation was particularly engineered for rapid release to ensure that bioactive AT-RvD1 was available during the critical early inflammatory phase. However, these findings remain preliminary, as Turner’s study is currently available as a preprint and has not yet undergone peer review [78].

Although the aforementioned studies investigated different SPM families and precursor molecules, collectively, they highlight the therapeutic potential of SPM-based strategies to enhance inflammation resolution [79]. However, most preclinical studies have been conducted in non-exercise injury models, limiting direct translation to EIMD contexts. In addition, variability in administration routes (e.g., systemic, intramuscular, topical) and treatment compositions (individual SPM molecules or intermediates) complicates the extrapolation of optimal treatment protocols. Table 1 summarizes the injury models, routes of administration, SPM molecules tested, dosing regimens, treatment timelines, and key biological outcomes across the preclinical studies discussed. Despite the promising regenerative and anti-inflammatory effects observed, future research is needed to determine whether these mechanisms can promote recovery following EIMD in humans.

### 4.2. Identification and Quantification of SPMs and Their Pathway Intermediates in Humans

While preclinical studies have established SPMs as critical mediators of inflammation resolution, their therapeutic potential ultimately depends on their endogenous presence, metabolism, and bioactivity. Advances in lipidomics, particularly LC-MS/MS and enzyme-linked immunosorbent assays (ELISAs), have enabled the identification and quantification of SPMs and their pathway intermediates in human biological samples. Bioactive levels of SPMs have been detected in various human fluids, including plasma, serum, human cord blood, synovial fluid, saliva, sputum, breast milk, and cerebrospinal fluid, as well as in tissues such as skeletal muscle [53], lymph nodes, and adipose tissue, across diverse age groups and health statuses. Calder has comprehensively reviewed SPM concentrations across different populations and tissues, reporting that baseline levels in healthy individuals are typically low or below detection limits (0.1 nM) [32]. Under non-inflammatory conditions, SPMs are generally present at nanomolar to picomolar concentrations [19,32,81] and are often undetectable in uninjured skeletal muscle [53].

### 4.3. Bioavailability Challenges in the EPA-DHA Conversion to SPMs

The bioavailability of SPMs derived from EPA and DHA depends on the accessibility of their precursor substrates. A key limiting step in lipid mediator biosynthesis is the liberation of PUFAs from membrane phospholipids, a process primarily regulated by secreted calcium-dependent phospholipase A_2_ (PLA_2_). Dietary intake of EPA and DHA increases the availability of free *n*-3 PUFAs, elevating concentrations of both SPMs and SPM-intermediates. In the absence of dietary intake, EPA and DHA largely remain sequestered within complex lipid structures, such as phospholipids, triglycerides, and cholesterol esters, requiring enzymatic release before SPM biosynthesis can occur [27,29,31,82,83,84,85].

Several studies have demonstrated that fish oil supplementation elevates plasma and serum SPMs and their intermediates across different durations and dosages. In short-term interventions (seven days), supplementation with 840 mg EPA + 600 mg DHA increased circulating SPM levels [27]. Moderate-term protocols (three weeks) using 1400 mg EPA + 1000 mg DHA similarly elevated SPM concentrations [28]. Long-term supplementation (three to twelve months) with either low (3.27 g/week) or high (13 g/week) EPA+DHA dosages also increased SPM production [29]. Notably, maternal *n*-3 PUFA supplementation during pregnancy (20 weeks) with 110 mg EPA + 280 mg DHA daily has been shown to influence SPM biosynthesis in both maternal circulation [30] and offspring [31]. Emerging evidence suggests that a threshold intake of EPA+DHA may be necessary to saturate phospholipid pools and optimize conditions for SPM synthesis. However, the precise dosages and concentrations required to achieve such an effect remain unclear [32].

While dietary EPA and DHA are fundamental for SPM production, enzymatic conversion efficiency influences SPM concentrations and contributes to variability across studies [29,31,32,83,86,87,88,89]. A comprehensive review by Calder [32] demonstrated that EPA supplementation leads to predictable dose-dependent increases in E-series resolvins (RvE1, RvE2, RvE3), whereas DHA supplementation fails to consistently elevate D-series resolvins, such as RvD1. This is particularly concerning given RvD1’s suggested role in muscle recovery, as reported in preclinical research [17].

Given these complexities, different marine-derived supplement formulations may help enhance SPM bioactivity and immune modulation. For example, Meganol D, a DHA-rich (75%) triglyceride formulation, has demonstrated superior efficacy in enhancing phagocytic capacity and vascular health compared to formulations with lower DHA content and fewer SPM intermediates [90]. This enhanced immune response was attributed to increased production of DHA-derived resolvins (e.g., RvD1, RvD2) [90]. Thus, Meganol D’s high DHA content may help overcome metabolic limitations of DHA conversion.

Considering the inconsistencies in SPM synthesis from DHA, future studies evaluating the efficacy of *n*-3 PUFA supplementation should account for baseline *n*-3 PUFA status and aim to optimize target SPM profiles rather than relying solely on EPA or DHA intake. Accordingly, SPM concentrations have been proposed as predictive biomarkers for *n*-3 therapeutic efficacy due to their ability to indicate enzymatic conversion efficiency and potential clinical benefits [90].

### 4.4. Analgesic Properties of SPMs and Their Intermediates

Beyond their role in immune modulation, SPMs and their intermediates show promise in pain management. In patients with arthritis, naturally occurring plasma SPM concentrations were negatively associated with Erythrocyte Sedimentation Rate (ESR), a clinical marker of systemic inflammation, while synovial fluid RvE2 concentrations were inversely associated with pain scores [26]. These findings suggest that higher endogenous SPM levels are linked to reduced inflammation and pain.

Emerging evidence also indicates that certain SPM intermediates, such as 17-HDHA, may independently modulate pain sensitivity. For example, 17-HDHA, a D-series resolvin intermediate, has been associated with enhanced pain tolerance in both healthy individuals and patients with osteoarthritis [91]. Importantly, these effects were independent of DHA, total *n*-3 levels, and AA levels [91], suggesting that the analgesic properties of 17-HDHA may rely more on efficient metabolic conversion rather than on traditional fish oil supplementation alone. As these findings support further investigation into the therapeutic potential of SPM intermediates in pain management, randomized controlled trials are warranted to determine whether direct administration of SPM intermediates effectively reduces pain.

## 5. Current Evidence, Applications, and Knowledge Gaps on SPM-Enriched Marine Oil

### 5.1. SPM-Enriched Marine Oil Supplementation in Humans

The identification of SPMs as key regulators of inflammation has sparked interest in their therapeutic applications through SPM-enriched marine oil supplements. These formulations combine SPM pathway intermediates (14-HDHA, 17-HDHA, and 18-HEPE) with low doses of the *n*-3 PUFAs EPA and DHA to enhance inflammation resolution and support muscle recovery. Unlike direct administration of isolated SPMs, SPM pathway intermediates bypass multiple upstream enzymatic conversion steps required in EPA and DHA metabolism; therefore, intermediates only require one additional enzymatic conversion to yield bioactive SPMs. Importantly, supplementation with pathway intermediates preserves endogenous regulatory control of SPM synthesis, offering a more physiological approach to inflammation resolution [17,19]. Despite the growing commercial availability of SPM-enriched marine oils, scientific investigation into their efficacy remains in its early stages.

Recent investigations demonstrate that SPM-enriched marine oil supplementation elicits rapid physiological effects on immune function and inflammation resolution. Within two hours of administration, serum and plasma SPM concentrations increase [18], with enhanced immune cell responses persisting for 24 h in healthy individuals [18,21,32]. A proposed mechanism involves the downregulation of cell adhesion molecules (CD11b, CD16, and CD41) on immune cells [22], a process that reduces neutrophil tissue infiltration and preserves muscle fiber integrity by modulating platelet–leukocyte interactions [92]. These findings parallel previous observations of peak induction of both naturally occurring pro-inflammatory and SPM pathway intermediates during early recovery from exercise, detected in both serum [58] and skeletal muscle [53] following unaccustomed resistance exercise.

To elucidate the clinical relevance of SPM-enriched marine oils, recent studies have investigated dose-dependent responses and short-term supplementation protocols in healthy individuals. Supplementation with 3 and 4.5 g significantly increased plasma SPM concentrations, whereas 1.5 g had no meaningful effect compared to placebo [18]. At higher doses (6 g), SPM levels peaked between 3 and 6 h post-administration, with higher concentrations detected in plasma versus serum [21]. In contrast to acute supplementation protocols, a 5-day regimen demonstrated sustained effects on inflammatory responses, including enhanced lipid mediator production and increased monocyte phagocytic activity [22]. These findings suggest that SPM supplementation may improve the body’s capacity to resolve inflammation over time. While short-term studies in healthy individuals have established basic pharmacokinetic profiles [18,21,22], the effects of supplementation during acute inflammatory conditions remain unexplored.

Emerging evidence from three human trials suggests therapeutic potential for SPM-enriched marine oil in pain management [20,23,25]. Given the mechanistic overlap between chronic low-grade localized inflammation (as observed in osteoarthritis and chronic pain) and the transient inflammation induced by EIMD, these findings raise the possibility that SPM pathway intermediates could support post-exercise recovery. Although no studies have directly investigated the role of SPM pathway intermediate concentrations in EIMD, pain-focused trials provide a rationale for exploring their potential to mitigate exercise-induced muscle soreness.

Across diverse study designs and populations, supplementation ranging from 500 mg to 2 g per day over four to twelve weeks consistently reduced chronic pain intensity and interference [20,23,25]. Notably, two studies implemented adaptive dosing strategies based on early symptom response [20,25]. One of these includes a four-week open-label trial in older adults, in which participants received 1.5 g daily, with mid-intervention dose adjustments at week two: those reporting a ≥2-point reduction in pain decreased their dose to 1 g, while those with ≤1-point improvement or worsening pain increased their dose to 2 g [20]. This study reported that 36% of participants experienced improved pain within just two weeks, although no significant changes in inflammatory biomarkers were observed at any timepoint (e.g., ESR and CRP) [20]. However, the absence of a placebo group and the open-label design limit the interpretability of these findings [20].

In addition, in a 12-week randomized controlled trial in individuals with osteoarthritis, participants received 2 g daily, with a protocol-defined dose reduction to 1 g at week six [25]. While reduced pain was observed during supplementation, benefits dissipated 12 weeks after intervention cessation [25], potentially suggesting the importance of continued supplementation for optimal outcomes. Lastly, a third study combining 500 mg of SPM-enriched marine oil with curcumin also reported improvements in pain and physical function [23]. However, due to the co-administration of dietary supplements, it remains unclear whether the observed benefits were primarily attributable to the SPMs, curcumin, or their combined effects.

Collectively, these findings suggest that SPM supplementation may be responsive to individual symptom trajectories and raise the possibility that dose personalization could enhance therapeutic efficacy. However, the preliminary nature of the current evidence, including the reliance on subjective measures, the absence of biomarker assessment in most studies (e.g., circulating SPMs, SPM pathway intermediates, or inflammatory markers), and the limited use of randomized controlled trials, underscores the need for future trials incorporating both mechanistic and functional outcomes. A summary of peer-reviewed studies investigating the effects of SPM-enriched marine oil supplementation on circulating SPMs, immune biomarkers, and pain outcomes in humans is presented in Table 2.

### 5.2. Dosage Variability in SPM-Enriched Marine Oils

Studies examining SPM-enriched marine oil supplementation reveal substantial variability in plasma SPM responses, largely due to differences in supplement composition. Among the eight human trials utilizing SPM-enriched marine oil to date, dosages are typically reported by total oil weight (e.g., capsule weight in grams) rather than by the content of active ingredients [18,19,20,21,22,23,25]. Fewer than half of these studies provide detailed quantification of key bioactive components, including EPA, DHA, and SPM pathway intermediates, such as 18-HEPE, 17-HDHA, and 14-HDHA [19,21,22]. When reported, SPM pathway intermediate content is often presented as a combined total of these three molecules [24], limiting the ability to assess their individual contributions. This lack of standardized reporting complicates comparisons across studies and raises uncertainty regarding whether the observed effects are primarily attributable to SPM intermediates, EPA, DHA, or the SPM-enriched marine oil formulation. Such heterogeneity is characteristic of early-stage supplementation research but presents challenges for identifying optimal dosing strategies. Although dose-dependent increases in plasma SPMs support the therapeutic potential of SPM-enriched marine oil, further investigation is needed to determine effective dosing protocols, treatment duration, and the specific contributions of individual lipid mediators.

### 5.3. SPM-Enriched Marine Oil and Recovery After Exercise-Induced Muscle Damage

**Figure 3:**
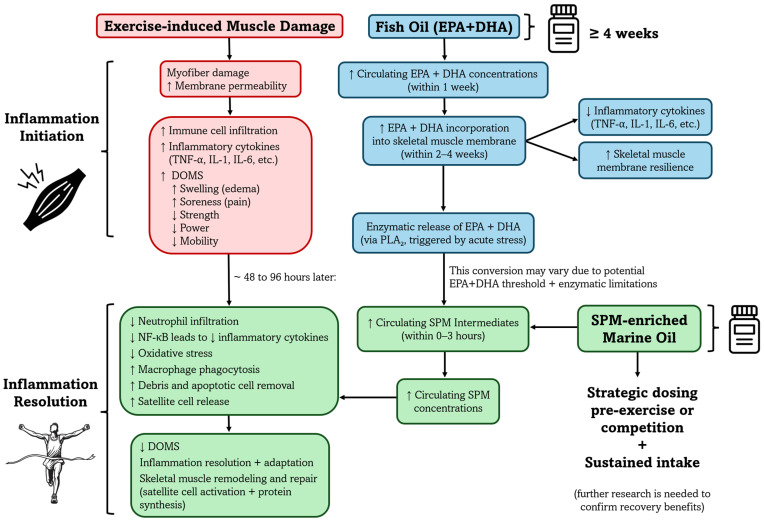
Proposed mechanisms by which SPM-enriched marine oil supplementation may support recovery following exercise-induced muscle damage (EIMD). Emerging evidence suggests that the anti-inflammatory effects of *n*-3 PUFAs are largely mediated through their enzymatic conversion into specialized pro-resolving mediators (SPMs). This conceptual model outlines the potential effects of SPM-enriched marine oil on key processes involved in inflammation resolution and tissue regeneration based on preclinical and mechanistic studies in inflammation and muscle injury contexts. These mechanisms may contribute to accelerated recovery of muscle function and pain reduction following eccentric or unaccustomed exercise. Arrows indicate direction of change: ↑ denotes an increase, and ↓ denotes a decrease. Abbreviations: EPA (eicosapentaenoic acid); DHA (docosahexaenoic acid); DOMS (Delayed Onset of Muscle Soreness); NF-kβ (nuclear factor kappa beta).

Despite the established role of SPMs in promoting inflammation resolution, the potential of SPM-enriched marine oil supplementation to enhance recovery from EIMD remains unexplored. Given SPMs’ ability to modulate acute inflammatory responses, supplementation may accelerate recovery by promoting timely resolution and improving performance outcomes. A conceptual model outlining the proposed mechanisms by which SPM-enriched marine oil may enhance recovery following EIMD is presented in Figure 3. However, human randomized controlled trials are needed to determine whether supplementation with SPM pathway intermediates can in fact attenuate impairments in muscle function and reduce pain following eccentric, unaccustomed exercise. Future investigations should aim to establish optimal supplementation protocols and clarify the mechanisms of SPM-mediated analgesic effects. In addition to biomarker assessments, future trials should incorporate functional outcomes such as maximal voluntary contraction, muscle soreness ratings, range of motion, and recovery of sport-specific performance metrics [93]. Standardized exercise protocols and validated recovery assessments will be critical for determining the clinical relevance of SPM-enriched marine oil supplementation in athletic and physically active populations.

### 5.4. Potential Applications in Physically Active and Trained Populations

Physically active individuals demonstrate superior inflammatory regulation compared to sedentary or less active individuals. Examples include rapid enzymatic responses (e.g., COX-2 upregulation within minutes of exercise initiation) [94], a more pronounced decrease in SPM pathway intermediate concentrations following exercise (suggested to reflect enhanced uptake for rapid inflammation resolution) [94], a peak in SPM production 24 h post-exercise (compared to 72 h in less active individuals) [94], lower resting inflammatory markers [95,96], and more efficient repair mechanisms following muscle-damaging exercise [97,98,99]. Consistent with these observations, preclinical research demonstrates that physically active mice exhibit enhanced inflammation resolution compared to sedentary controls, characterized by increased macrophage phagocytic activity, elevated RvD1 levels, and accelerated neutrophil clearance following an acute inflammatory challenge [100].

Thus, in conditioned populations where tissue damage is attenuated and recovery is already accelerated [93], SPM-enriched marine oil supplementation may not address impaired resolution but rather optimize existing recovery processes through targeted molecular actions, thereby potentially accelerating a return to peak performance after strenuous exercise or muscle injury. However, no studies have yet demonstrated the effectiveness of SPM pathway intermediate concentrations found in commercially available supplements for enhancing exercise recovery, nor have studies characterized the conversion rates of EPA and DHA to SPM pathway intermediates or from SPM intermediates to bioactive SPMs. Addressing these questions is critical for clarifying whether SPM-focused strategies offer tangible advantages for mitigating EIMD in humans.

### 5.5. Contextualizing SPM-Enriched Marine Oil Within the Broader Fish Oil Literature

While this review focuses on the emerging potential of SPM-enriched marine oil, it is important to recognize that conventional fish oil supplementation elevates circulating SPM concentrations in humans across various dosing protocols and durations [27,28,32]. However, no studies to date have directly compared the efficacy of SPM-enriched marine oil with traditional fish oil in modulating SPM levels or enhancing recovery following EIMD. Given that EPA and DHA serve as substrates for SPM biosynthesis, it is plausible that, over time, the cumulative effect of high-dose EPA+DHA supplementation could lead to comparable tissue concentrations of bioactive SPMs, such as those achieved acutely with SPM-enriched formulations [42,43]. By contrast, SPM-enriched formulations containing SPM pathway intermediates may bypass certain enzymatic bottlenecks, leading to a more rapid but potentially transient increase in circulating SPMs [18,21,22]. Although this acute increase may contribute to pain modulation and immune cell reprogramming, it remains unclear whether such effects are sufficient to improve EIMD recovery in the absence of sustained tissue-level incorporation of EPA and DHA. Therefore, SPM-enriched marine oil may complement—but not replace—the established benefits of long-term fish oil supplementation, and future comparisons are warranted to delineate their respective contributions to inflammation resolution and muscle regeneration.

### 5.6. Analytical Challenges and Methodological Considerations in Quantifying SPMs in Humans

Despite increasing interest in the biological roles of SPMs, their accurate quantification remains technically challenging. These challenges arise from their low endogenous concentrations (picogram-to-nanogram range), structural similarity to other lipid mediators, and susceptibility to matrix interference. Liquid chromatography–tandem mass spectrometry (LC-MS/MS) is considered the gold standard due to its high sensitivity and specificity, enabling the detection of SPMs at low concentrations [101]. However, it requires rigorous validation, including adherence to standard signal-to-noise thresholds, to avoid overestimation or false-positive detection. Furthermore, pre-analytical variables also affect quantification. For example, resolvins quickly degrade during repeated freeze–thaw cycles, and plasma, preferably processed rapidly and stored at −80 °C, is generally favored over serum as it minimizes platelet activation [102]. 

Recent methodological debate has further underscored the need for technique standardization. O’Donnell et al. highlighted inconsistencies in applying detection criteria that may overestimate SPM levels [103]. In response, Dalli and Gomez reanalyzed their data using independent validation techniques (orthogonal validation) and defended the biological relevance of their findings [51]. While several laboratories have confirmed the presence and bioactivity of SPMs in human samples [50], these discussions emphasize the need for transparent and standardized protocols. Key recommendations include the use of authentic reference standards, retention time confirmation, and the verification of fragmentation patterns. Without these precautions, quantification errors may compromise the interpretation of SPM data. 

Additionally, enzyme-linked immunosorbent assays (ELISAs) have also been used to quantify certain SPMs and have shown strong correlation with LC-MS/MS (r = 0.93) [104]. However, ELISA presents limitations, including cross-reactivity between structurally similar mediators (e.g., RvD1 vs. RvD2) and variability in sensitivity across kits. To date, serum or plasma RvD1 has been measured via ELISA in a limited number of studies, primarily involving clinical populations with chronic or inflammatory conditions rather than healthy or physically active individuals [105,106,107,108,109,110,111,112]. Together, these considerations highlight the importance of methodological rigor and detailed reporting when interpreting SPM concentrations.

## 6. Conclusions

SPM-enriched marine oil supplementation presents a promising strategy to enhance EIMD recovery by promoting inflammation resolution. Unlike traditional *n*-3 PUFA supplementation, which requires two to four weeks for initial incorporation, and at least four weeks to significantly alter skeletal muscle fatty acid composition, SPM-enriched marine oil formulations may accelerate recovery by supporting immediate endogenous SPM synthesis. Current evidence demonstrates rapid biological effects, including increased plasma concentrations and modulated immune responses within hours of administration. Despite these promising findings, several critical challenges remain, such as the following: (1) functional and clinical outcomes remain undefined, (2) optimal formulations and dosing protocols have not been established, and (3) randomized controlled trials are lacking. While preliminary data from populations experiencing chronic pain suggests therapeutic potential, no studies to date have investigated the effects of SPM-enriched marine oil supplementation on recovery from exercise. Future randomized controlled trials incorporating both mechanistic biomarkers and functional performance outcomes are needed to clarify the role of SPM-enriched marine oil supplementation in optimizing recovery and enhancing performance in sports and exercise contexts.

Summarizing the main findings presented in this narrative review, we highlight the following teaching points:

- SPMs are endogenously synthesized from EPA and DHA via enzymatic conversion and actively coordinate inflammation resolution, distinguishing their role from agents that merely suppress inflammation, such as NSAIDs.
- SPM-enriched marine oil supplements contain low concentrations of EPA, DHA, and SPM pathway intermediates such as 14-HDHA, 17-HDHA, and 18-HEPE; however, optimal concentrations remain undefined, and it is unclear whether observed increases in plasma SPM concentrations are primarily driven by EPA+DHA content or by the direct contribution of the SPM pathway intermediates.
- Dosages of 3 and 4.5 g of SPM-enriched supplements, but not 1.5 g, have been shown to elevate plasma SPM concentrations within two hours; however, whether this acute increase translates into meaningful exercise recovery benefits remains unknown.
- Individual responses to SPM supplementation vary significantly, but daily doses of 1–2 g, often initiated at 2 g and tapered to 1.5 or 1 g based on early pain improvement, have demonstrated analgesic effects in chronic pain conditions over treatment periods of 4 to 12 weeks.
- The efficacy of SPM supplementation for exercise recovery remains theoretical and requires validation through randomized controlled trials, with no studies directly comparing SPM-enriched marine oil to traditional fish oil (EPA+DHA) supplementation.

## Figures and Tables

**Table 1:** Summary of preclinical studies investigating the effects of specialized pro-resolving mediators (SPMs) and SPM intermediates on muscle regeneration, tissue repair, pain, and recovery outcomes.

Injury Model	Administration	Molecule	Regimen	Timeline	Key Outcomes
Acute Muscle Injury (Mice) [17]	Systemic	RvD1	100 ng/day	5 min post-injury to 14 days	↓ IL-6 expression ↓ TNF-α expression ↓ Neutrophil infiltration ↑ Myofiber CSA ↑ Muscle strength recovery ↑ M2 macrophages
Muscle Regeneration Impairment (Mice) [59]	Intramuscular	RvD2	4 µg/kg	Single dose, 3 days post-injury	↑ M2 macrophages ↑ Force recovery ↑ Muscle mass
Volumetric Muscle Loss (Mice) [78]	Topical	AT-RvD1	100 ng/day	Following injury (24 h gradual release)	↑ Maximal isometric torque recovery
Arthritis (Rats) [80]	Systemic	17[R]-HDHA or AT-RvD1	300 ng/day	4–5 days treatment (17[R]-HDHA once daily for 5 days; AT-RvD1 twice daily for 4 days)	↓ Pain ↓ Joint stiffness ↓ IL-1β expression ↓ TNF-α expression
Osteoarthritis (Rats) [79]	Systemic	17[R]-HDHA	300 ng/day	Single dose and repeated treatment every other day for 2 weeks	↓ Pain (rapid onset within 1 h, lasting up to 7 days post-treatment)
Periodontitis (Rabbits) [77]	Topical	RvE1	4 µg/site	3 times per week for 6 weeks	↓ IL-1β expression ↓ CRP expression ↑ Bone and soft tissue restoration

Arrows indicate direction of change: ↑ denotes an increase, and ↓ denotes a decrease. Abbreviations: RvD1 (Resolvin D1); RvD2 (Resolvin D2); AT-RvD1 (Aspirin-Triggered Resolvin D1); 17[R]-HDHA (17[R]-Hydroxy-docosahexaenoic acid); CRP (C-Reactive Protein), IL (interleukin); TNF-α (tumor necrosis factor-alpha); CSA (cross-sectional area); M2 (pro-resolution macrophages).

**Table 2:** Summary of peer-reviewed studies on the effects of marine oil supplementation on circulating specialized pro-resolving mediators (SPMs), immune biomarkers, and pain outcomes.

Ref.	Sample (*n*)	Population	Dosage	Duration	Circulating SPMs	Immune Biomarkers	Pain Outcomes	Limitations
[22]	20	Healthy + patients with PAD	1.5, 3, and 6 g	5 days + 9 days washout	↑	Enhanced phagocytosis Reduced Biomarkers (TNF-α and MCP-1)	-	Open label
[18]	22	Healthy adults	1.5, 3 and 4 g	Single dose	↑	Reduced adhesion molecule expression + increased phagocytosis	-	-
[20]	44	Adults with chronic pain	W 0–2: 1.5 g W 3–4: 1 g or 2 g (based on pain scores improvement)	4 weeks	-	No change (hs-CRP and ESR)	↓ pain intensity ↓ pain interference (PROMIS-43)	Open label, no placebo
[19]	23	Adults with obesity	2 g	28–30 days	↑	IgG concentrations decreased upon B-cell activation	-	No placebo
[23]	29	Adults with mild or moderate pain	0.5 g of enriched marine oil + 0.5 g curcumin	60 days	-	-	↓ total pain ↓ pain intensity ↓ pain severity (SF-MPQ)	No placebo
[21]	10	Healthy adults	6 g (60 mL)	Single dose	↑	-	-	-
[25]	51	Adults with knee OA	W 0–6: 2 g W 7–12: 1 g	12 weeks	-	-	↓ pain (OMERACT-OARSI score)	-
[24]	53	Adults with post-COVID syndrome	0.5, 1.5, and 3 g	12 weeks	↑ *	-	-	Lack of clinical outcomes

* Significant increases in SPM pathway intermediates were observed, although changes in SPMs remained unclear. Arrows indicate direction of change: ↑ denotes an increase, and ↓ denotes a decrease. Abbreviations: TNF-α (tumor necrosis factor-α); MCP-1 (monocyte chemoattractant protein-1); hs-CRP (High-Sensitivity C-Reactive Protein); ESR (Erythrocyte Sedimentation Rate); W (week); PROMIS-43 (Patient-Reported Outcomes Measurement Information System-43 Profile); SF-MPQ (the Short-Form McGill Pain Questionnaire); OMERACT-OARSI (a combination between the Outcome Measures in Rheumatology [OMERACT] and the Osteoarthritis Research Society International [OARSI]).

## Data Availability

Data sharing is not applicable to this article. No datasets were generated or analyzed as it is a narrative review of existing literature.

## Abbreviations

The following abbreviations are used in this manuscript:

14-HDHA	14-Hydroxy-Docosahexaenoic Acid
17-HDHA	17-Hydroxy-Docosahexaenoic Acid
17(R)-HDHA	17(R)-Hydroxy-docosahexaenoic acid
18-HEPE	18-Hydroxy-Eicosapentaenoic Acid
14-HpDHA	14-Hydroxyperoxydocosahexaenoic acid
17-HpDHA	17-Hydroxyperoxydocosahexaenoic acid
AA	arachidonic acid
ALA	alpha-linoleic acid
AT-RvD1	Aspirin-Triggered Resolvin D1
CD11b	Cluster of Differentiation 11b
CD16	Cluster of Differentiation 16
CD41	Cluster of Differentiation 41
CD163+	Cluster of Differentiation 163-positive
COX	cyclooxygenase
COX-2	cyclooxygenase-2
CRP	C-Reactive Protein
DHA	docosahexaenoic acid
EIMD	exercise-induced muscle damage
ELISA	enzyme-linked immunosorbent assay
EPA	eicosapentaenoic acid
ESR	Erythrocyte Sedimentation Rate
IGF-1	Insulin-Like Growth Factor 1
IL-1β	interleukin 1 beta
IL-6	interleukin 6
LA	linoleic acid
LC-MS/MS	liquid chromatography–tandem mass spectrometry
LOX	lipoxygenase
LTs	leukotrienes
M1	macrophage phenotype 1
M2	macrophage phenotype 2
MaR1	Maresin 1
NF-κB	nuclear factor kappa beta
NSAIDs	non-steroidal anti-inflammatory drugs
n-3 PUFAs	omega-3 polyunsaturated fatty acids
n-6 PUFAs	omega-6 polyunsaturated fatty acids
Pax7+	paired box 7-positive
PGI_2_/PGI_3_	Prostacyclin I2/I3
PGs	prostaglandins
PLA_2_	Phospholipase A2
PD1	Protectin D1
ROS	reactive oxygen species
RvD1-RvD6	Resolvins D1 to D6 (DHA-derived)
RvE1-RvE3	Resolvins E1 to E3 (EPA-derived)
SPMs	specialized pro-resolving mediators
TNF-α	tumor necrosis factor-alpha
TXs	thromboxanes
